# Changes in Intake and Major Food Sources of Carotenoids among U.S. Adults between 2009–2018

**DOI:** 10.3390/metabo14010013

**Published:** 2023-12-24

**Authors:** Kijoon Kim, Matthew P. Madore, Ock K. Chun

**Affiliations:** 1 Department of Nutritional Sciences, University of Connecticut, Storrs, CT 06269, USA; drkijoon@sookmyung.ac.kr (K.K.); matthew.p.madore@uconn.edu (M.P.M.); 2 Department of Food and Nutrition, Sookmyung Women’s University, Seoul 04310, Republic of Korea; 3 Kim Kijoon BOM Clinic, Seoul 05554, Republic of Korea

**Keywords:** carotenoids, vegetables, phytochemicals, fruit, vitamin A adequacy

## Abstract

Large epidemiologic studies suggest that diets rich in total or specific carotenoids are associated with a reduced risk of many chronic diseases. However, there are few studies characterizing trends in dietary carotenoid sources and intake among subgroups of the US adult population in the previous decade. This study aimed to assess these trends using data from 22,339 adults who participated in NHANES 2009–2018 cycles. Carotenoid intake and major food sources were calculated by linking food consumption data from the 24 h diet recall to an FNDDS 2009–2018 and the USDA’s National Nutrient Database for Standard Reference (Release 28). Among US adults, mean (SE) dietary carotenoid intake was 9687.1 (158.0) mcg/day, and total intake was highest in men, non-smokers, moderate alcohol consumers, supplement users, and those with normal BMI, a PIR ≥ 1.85, and whose physical activity level was considered vigorous (*p* < 0.05). Carotenoid intake has gradually decreased over the past decade (*p*-trend: 0.097), especially among White adults (*p*-trend < 0.05), males (*p*-trend: 0.062), and those with a PIR of 1.0–1.3 (*p*-trend: 0.051), as have estimated rates of vitamin A adequacy. Tomatoes, carrots, and spinach were major food sources of carotenoids, and consumption of carrots and tomatoes decreased, while the consumption of lettuce, spinach, and salsa increased from 2009 to 2018. Our results warrant further studies investigating the consequences of the decreased tendencies of carotenoid intake on chronic disease risk, especially focusing on population subgroups exhibiting low or decreasing trends of carotenoid intake status.

## 1. Introduction

Carotenoids are a group of natural pigments found in various fruits and vegetables that have been associated with numerous health benefits. Carotenoids act as antioxidants and provitamin A, contributing to their significant role in promoting health [1]. Epidemiological studies have consistently demonstrated that higher dietary intake of carotenoids is associated with reduced risks of chronic diseases, such as cardiovascular disease [2,3,4,5], cancer [6,7,8,9,10], and age-related eye diseases [11], as well as improved immune function [12], reductions in inflammation [13], and enhanced cognitive functions [14]. Numerous studies have explored the association between carotenoid intake and health outcomes, leading to a growing interest in understanding the dietary sources and trends in carotenoid intake among different populations [15,16,17,18].

Several studies have investigated carotenoid intake among U.S. adults; however, there is limited research characterizing major carotenoid sources and trends in intake over time among various population subgroups in the recent decade [19]. Furthermore, previous studies [20] have varied in their methodologies for assessing carotenoid intake, making it challenging to compare findings across studies and accurately estimate trends over time. Moreover, the dietary landscape has changed over the past decade, with shifts in food availability, accessibility, and consumption patterns [21]. These changes may have influenced carotenoid intake trends among different subgroups of the U.S. adult population. Therefore, there is a need for updated and comprehensive research to provide a current understanding of major carotenoid sources and trends in intake among diverse population subgroups.

In the global context, studies in various countries have demonstrated diverse patterns in carotenoid intake. While studies in five European countries [22] showed significant variations in median carotenoid intake across nations and emphasized the role of traditional dietary sources, studies in Asia, particularly Singapore [23], and in South America, especially Brazil [15], reported mean intake levels, highlighting the influence of dietary patterns and socioeconomic factors on carotenoid consumption. With their varied methodologies, these international studies offer valuable perspectives that complement our understanding of carotenoid intake in the U.S. population.

Dietary vitamin A is obtained through two primary sources: preformed vitamin A, including retinol and retinyl esters, and provitamin A carotenoids, notably beta-carotene, alpha-carotene, and beta-cryptoxanthin, which contribute to overall vitamin A intake. Vitamin A deficiency (VAD) is one of the most common nutrient deficiencies in the world, following iron [24]. VAD is associated with numerous health risks, including increased susceptibility to infectious diseases [25], impaired immune function [26], growth and developmental delays in children [27], and vision loss [28]. The most pronounced manifestations of VAD are observed among young children and pregnant women residing in low-income countries [29]. The Food and Agriculture Organization (FAO) also expresses significant concerns about the eye health of low-income countries, and the extent of VAD varies greatly among different nations [30,31]. While the United States has a lower prevalence of severe VAD compared to other countries, there is still a significant proportion that falls short of the Recommended Dietary Allowance (RDA) [19]. Furthermore, there appears to be substantial variation in VAD among subgroups such as race, age, income levels, and fruit and vegetable intake disparities. Therefore, it is crucial to understand the vulnerability of specific subgroups to inadequate vitamin A intake, given the critical role of carotenoids as a source of vitamin A.

The objective of this study was to estimate carotenoid intake levels and changes in intake as well as to identify major food sources of carotenoids among U.S. adults between 2009 and 2018 using data from the National Health and Nutrition Examination Survey (NHANES). Understanding the major food sources and trends in carotenoid intake among U.S. adults is critical for informing public health efforts to promote healthy dietary patterns. This study fills a gap in the literature by providing updated and comprehensive data on carotenoid intake and major food sources among diverse subpopulations of U.S. adults over the past decade. The findings of this study can contribute to the development of targeted interventions and policies to improve carotenoid intake and overall health outcomes in the U.S. population.

## 2. Methods

### 2.1. Study Population

This cross-sectional study used 22,339 US adults aged 19 years and older from the NHANES 2009–2018 cycles. For this analysis, those with dietary recalls coded as unreliable or incomplete (*n* = 683), those under the age of 19 (*n* = 13,822), and women who were pregnant (*n* = 253) or breastfeeding (*n* = 154) were excluded.

### 2.2. Dietary Intake of Carotenoids

This analysis used What We Eat in America (WWEIA) data from the NHANES 2009–2018 cycles, which includes two days of dietary intake data for each participant. These data were estimated from 24 h dietary recall interviews conducted by trained interviewers using the USDA Automated Multiple Pass Method. The first dietary recall interviews are conducted in person by trained dietary interviewers in the Mobile Examination Center (MEC). The second dietary recall is collected by telephone and is scheduled 3 to 10 days later. In supplement Table S1, estimates of energy-adjusted nutrient intakes obtained using the density method, in which nutrient intakes are calculated per 1000 kcal, are presented.

### 2.3. Estimation of Carotenoid Intake and Major Food Sources

Dietary carotenoid intake was estimated from two 24 h dietary recalls collected during each of the 5 NHANES cycles between 2009 and 2018. The average carotenoid intake of each participant was calculated by averaging their intake across the two 24 h dietary recalls. This estimation included individual carotenoids such as alpha-carotene, beta-carotene, beta-cryptoxanthin, lycopene, lutein + zeaxanthin, and the total carotenoid intake. In addition to carotenoid intake, vitamin A and retinol intakes were derived from NHANES dietary data. Provitamin A estimation involved summing the intakes of alpha-carotene, beta-carotene, and beta-cryptoxanthin. Throughout this calculation, unit conversions were consistently applied: One microgram of Retinol Activity Equivalents (mcg RAE) corresponds to 1 mcg of retinol, 2 mcg of supplemental beta-carotene, 12 mcg of dietary beta-carotene, or 24 mcg of dietary alpha-carotene or beta-cryptoxanthin [32]. The major food sources of carotenoids were estimated using NHANES dietary data and the USDA’s Food and Nutrient Database for Dietary Studies (FNDDS) version 5.0 (2009–2010) [33], the FNDDS 2011–2012 [34], the FNDDS 2013–2014 [35], the FNDDS 2015–2016 [36], and the FNDDS 2017–2018 [37]. FNDDS is utilized as a resource for determining nutrient values in NHANES, and it relies on the nutrient information available in the USDA National Nutrient Database for Standard Reference (SR) [38]. FNDDS contains information on the link between foods in NHANES and SR items, enabling the estimation of carotenoid intake by summing the carotenoid content of each food item reported in the dietary recall. Using the FNDDS database, which includes recipes for foods, we identified the carotenoid content of each component in mixed dishes. We aggregated these to calculate the total carotenoid intake from each food source. This method allowed for the precise identification of major food sources of carotenoids by accounting for the carotenoids present in both single-food items and composite foods. Regarding the food group categories, beans included peas, lentils, cowpeas, and lupins. Tomato products included pasta, such as spaghetti sauce, marinara sauce, tomato paste, and tomato puree; pepper included pimento but excluded chili powder and chili; pasta included lasagna, spaghetti, ravioli, tortellini, and macaroni. Cereals included cereals that were ready-to-eat and cereal bars. Also, pickles included pickle relish and other sauces included barbecue sauce, steak sauce, and cocktail sauce but excluded gravy, salsa, pepper, guava, and chili. A final list of 107 foods, meticulously chosen for their high carotenoid concentrations, was created by selecting 30 individual food items per carotenoid and removing duplicates, forming distinct food group categories for analysis.

### 2.4. Statistical Analysis

Statistical analyses were conducted with SAS software, version 9.4 (SAS Institute Inc., Cary, NC, USA), using SAS survey procedures and the appropriate weight, strata, and cluster variables to account for the complex survey design used by NHANES. The mean and median of the carotenoid intake were calculated for participants with various sociodemographic and lifestyle characteristics. *p*-values for differences in carotenoid intake between subgroups were obtained from ANOVA and t-tests. The Cochran–Armitage test for trend was used to assess trends in vitamin A adequacy rate over 10 years. The *p*-value for the trend in carotenoid intake over the 10 years was determined using multivariable logistic regression and was adjusted for age, gender, ethnicity, and PIR. When conducting gender-specific analyses, gender was excluded from the adjustment variables. To provide descriptive statistics, participants were classified based on their poverty income ratio (PIR) into three groups: PIR < 1.3, 1.3 ≤ PIR < 1.85, and PIR ≥ 1.85. Alcohol consumption was defined as either no consumption (0 drinks), moderate consumption (no more than two drinks/day for men and no more than one drink/day for women), or heavy consumption (more than two drinks/day for men and more than one drink/day for women), based on the number of drinks of any type of alcoholic beverage participants reported drinking per day [39]. Smokers were classified as current smokers if they had smoked at least 100 cigarettes in their lifetime and smoked some days or every day, non-smokers if they had smoked fewer than 100 cigarettes in their lifetime, and former smokers if they had smoked at least 100 cigarettes in their lifetime but had reported quitting by the time of the interview. Physical activity was quantified as a metabolic equivalence of tasks (MET), calculated based on weekly minutes of walking/bicycling and moderate/vigorous recreational activities. MET-min/week was determined by multiplying weekly minutes of activities by the assigned MET values [40]. Subjects who reported no walking/bicycling or moderate/vigorous recreational activities were classified as inactive. All *p*-values reported are two-sided (α = 0.05).

## 3. Results

Among US adults, the mean dietary carotenoid intake was 9687.1 mcg/day (Table 1). Total carotenoid intake was highest in men, non-smokers, moderate alcohol consumers, supplement users, those with normal BMI (18.5–24.9), those with a poverty-income ratio (PIR) ≥1.85, and those with vigorous physical activity (*p* < 0.05). As for the age groups among each sex, total carotenoid intake was highest in men aged 31–50 years and women aged 51–70 years. When carotenoid consumption was adjusted for energy intake, total carotenoid intake was found to be greater with age and higher among women, non-smokers, supplement users, those with normal BMI (18.5–24.9), those with a poverty-income ratio (PIR) ≥1.85, and those with vigorous physical activity (*p* < 0.05) (Supplement Table S1).

**Figure 1:**
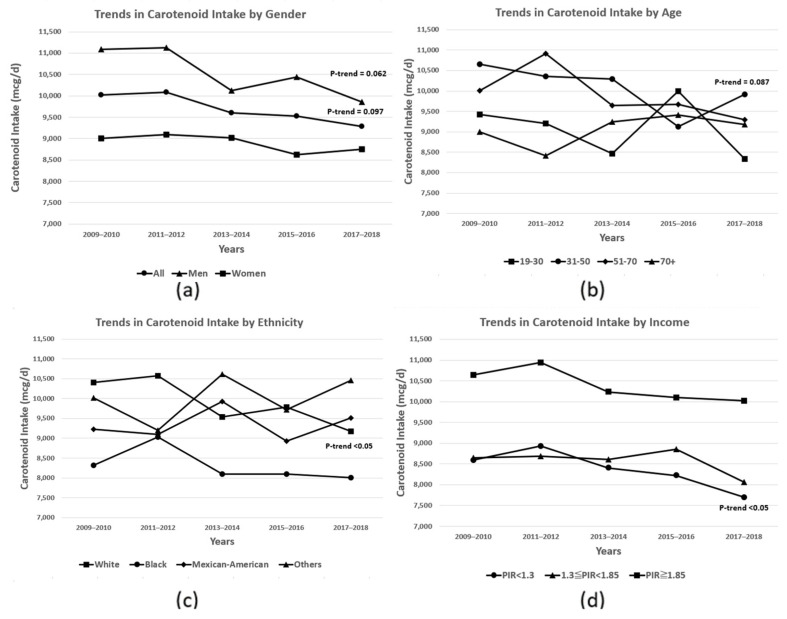
Trends in total dietary carotenoid intake in US adults aged 19+ y between 2009 and 2018 by (**a**) gender, (**b**) age, (**c**) ethnicity, and (**d**) income. Data are based on food consumption data from the NHANES 2009–2018 (n = 22,339). The *p*-value for the trend in carotenoid intake over the 10 years was determined using multivariable logistic regression and was adjusted for age, gender, ethnicity, and PIR. When conducting gender-specific analyses, gender was excluded from the adjustment variables.

Total carotenoid intake has gradually decreased over the past decade (*p*-trend: 0.097), especially in White adults (*p*-trend < 0.05), males (*p*-trend: 0.062), those aged 31–50 years (*p*-trend: 0.087), and those with PIR <1.3 (*p*-trend < 0.05) (Figure 1). Over the past 10 years, the trend in individual carotenoids intake has shown different patterns according to sociodemographic characteristics (Supplement Table S2). Additionally, vitamin A adequacy rates have decreased slightly over the past decade (Table 2), with decreases in retinol, α-carotene, and lycopene intake likely driving this reduction (Table 3). This trend was observed for most subgroups. However, there was an increase in adequacy rates among Mexican-Americans, other ethnicities, and those aged 70 years and older over 10 years (Table 2). While there have been no significant changes in the intake of provitamin A, a significant decrease was observed in retinol intake over the 10-year period. Among the provitamin A carotenoids, only the intake of alpha-carotene has declined over the past decade, while the intake of beta-carotene and beta-cryptoxanthin has remained relatively unchanged (Table 3, Supplement Table S3).

**Figure 2:**
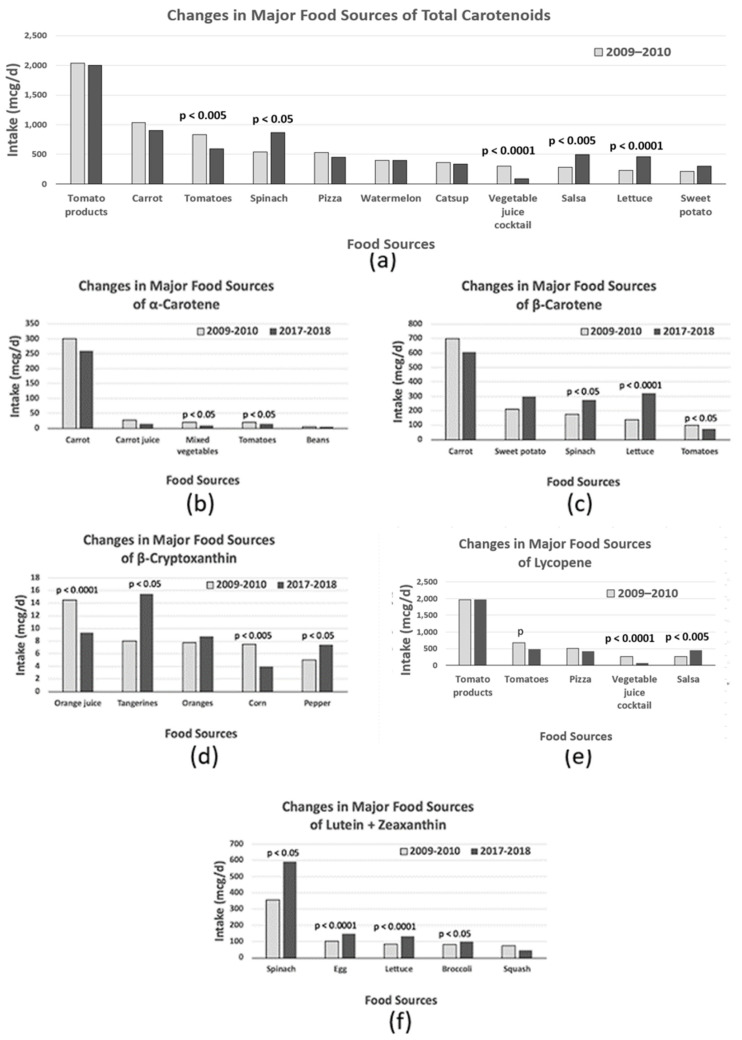
Changes in major carotenoid food sources between 2009–2010 and 2017–2018: A comparative analysis of (**a**) total carotenoids, (**b**) alpha-carotene, (**c**) beta-carotene, (**d**) beta-cryptoxanthin, (**e**) lycopene, and (**f**) lutein + zeaxanthin. Data are based on food consumption data from the NHANES 2009–2010 and 2017–2018 (*n* = 9244). *p*-values for differences in carotenoid intake between 2009–2010 and 2017–2018 were obtained from *t*-tests.

The major food sources of total carotenoid intake among US adults were mainly tomato products, followed by carrots, tomatoes, spinach, pizza, watermelon, ketchup, vegetable juice cocktail, salsa, lettuce, and sweet potatoes (Table 4). Over the past decade, significant decreases were observed in total carotenoid intake from tomatoes and vegetable juice cocktails, while significant increases were observed in total carotenoid intake from spinach, salsa, and lettuce (Figure 2). When examining the primary food sources of individual carotenoid intake, the major food sources of alpha-carotenoid were mainly carrots, followed by carrot juice, mixed vegetables, and tomatoes. Intake of alpha-carotenoid from mixed vegetables and tomatoes decreased significantly over 10 years. The major food sources of beta-carotenoid intake were carrots, sweet potatoes, spinach, lettuce, and tomatoes. Intakes from spinach and lettuce increased, and intakes from tomatoes decreased over the past 10 years. The major food sources of beta-cryptoxanthin intake were orange juice, tangerine, oranges, corn, and pepper. In the past 10 years, the intake of tangerine and pepper increased, while the intake of orange juice and corn decreased. The major food sources of lycopene intake were tomato products, tomatoes, pizza, vegetable juice cocktails, and salsa. Over the last 10 years, intake from tomato and vegetable juice cocktails decreased, and intake from salsa increased. The major food sources of lutein and zeaxanthin intake were spinach, eggs, lettuce, broccoli, and squash, and intakes from spinach, egg, lettuce, and broccoli increased significantly over the past decade.

## 4. Discussion

In this study, significant differences in carotenoid intake among sociodemographic subgroups of US adults stratified by characteristics such as gender, BMI, household income, alcohol consumption, smoking, physical activity, and supplement intake were observed. Considering that vegetables, including tomatoes, are the primary food sources of carotenoids, these findings are consistent with previous research suggesting substantial variations in vegetable consumption across sociodemographic subgroups [41,42,43]. Additionally, according to this analysis, carotenoid intake was significantly higher in males compared to females. Individuals aged 31–50 exhibited the highest intake within different age groups, while those aged 70 years and older had the lowest intake. However, after adjusting for energy intake, it was observed that females had significantly higher carotenoid intakes than males, and carotenoid intake increased with age. Seeing that energy adjustment is associated with diet quality, these results are consistent with previous research indicating an association between vegetable consumption and diet quality [44], as well as findings that females and older individuals tend to have better diet quality [45].

While observing the trend from 2009 to 2018, a marginally significant decline in total carotenoid intake among US adults was noted. Specifically, this study identified a marginally significant decrease in total carotenoid intake among males and individuals aged 31–50 and among White and low-income participants. While a previous study [19] by Han et al. based on NHANES 2003–2016 data reported an increasing trend in alpha-carotene intake, the present study observed the contrast; there was a trend toward decreased alpha-carotene intake. However, when analyzing different time periods, Han et al. demonstrated a consistent increase in alpha-carotene intake from 2003–2004, an apparent peak in 2011–2012, and a gradual decline following 2012. The trends observed in the current study, which investigated intakes from 2009–2010 and onwards, align with the decreasing pattern seen in the later years included in the previous study, suggesting that the apparent disparity in the overall trend can be attributed to differences in the research periods. In this study, trends in intakes of beta-carotene and lutein + zeaxanthin were consistent with previous research over a similar time period.

Certain carotenoids, such as α-carotene, β-carotene, and β-cryptoxanthin, have a clear function firmly linked to health outcomes, notably their provitamin A activity and role in preventing VAD [46]. This is supported by the findings of Böhm et al., demonstrating the conversion of α-carotene, β-carotene, and β-cryptoxanthin to retinol-equivalent vitamin A activity and recommendations for carotenoid intake [47]. Therefore, a requirement for carotenoids based upon vitamin A activity must be established in concert with the Dietary Reference Intakes (DRIs) assessment for vitamin A. Although no DRIs are proposed for β-carotene or other carotenoids at the present time, existing recommendations for increased consumption of carotenoid-rich fruits and vegetables are supported. The current study found a gradual decline in the vitamin A adequacy rate over the past decade, from 27.8% to 25.5%, consistent with previous research findings [19]. While there have been no significant changes in the intake of provitamin A, a significant decrease was observed in retinol intake.

The changes in total and individual carotenoid intake over the past decade indicate shifts in the consumption patterns of key carotenoid-rich foods among adults in the United States during this period. In this study, total carotenoid intake exhibited a declining trend over the past 10 years. Although there were significant increases in spinach, salsa, and lettuce consumption, there were larger decreases in intakes of tomatoes and vegetable juice cocktails. These changes are consistent with the observed 10% decrease in per capita consumption of tomatoes and 5% and 24% increases in per capita consumption of spinach and lettuce in 2017–2018 compared to 2009–2010, according to USDA ERS food availability data [48]. Examining the changes in the major food sources of individual carotenoids, this study found there was a decline in the consumption of alpha-carotene-rich foods, including carrots, carrot juice, mixed vegetables, and tomatoes, which may explain the slight decrease in alpha-carotene intake in 2017–2018 compared to 2009–2010. Furthermore, in this study, it was observed that the intake of major food sources of beta-carotene, such as carrots and tomatoes, was lower in 2017–2018 compared to 2009–2010, while the consumption of spinach and lettuce were greater, resulting in a slightly greater mean intake of beta-carotene intake in 2017–2018 compared to 2009–2010.

In this study, the intake of beta-cryptoxanthin did not show a significant change in 2017–2018 compared to 2009–2010. However, it was observed that there was a decline in the consumption of major food sources such as orange juice and corn, whereas tangerines and pepper consumption increased. These findings are also consistent with the USDA ERS food availability data [48], which indicates a respective 35% and 20% decrease in per capita consumption of orange juice and corn in 2017–2018 compared to 2009–2010, while intake of tangerines/tangelos and chili pepper demonstrated respective increases of 72% and 12%. With regard to the diversity of food carotenoid sources, this study found that over 80% of total carotenoid intake during the past decade was predominantly obtained from the top 20 major food sources. However, the contribution of the top 10 food sources of alpha-carotene to total intakes decreased from 95% in 2009–2010 to 78% in 2017–2018, suggesting a more diverse range of foods have contributed to total alpha-carotene consumption in recent years. In contrast, the proportion of beta-carotene and lutein + zeaxanthin intake supplied by the top 10 major food sources increased from 70% and 59% in 2009–2010 to 77% and 73% in 2017–2018, respectively, indicating a reduction in the diversity of food sources of beta-carotene and lutein + zeaxanthin.

Our findings on the carotenoid intake among U.S. adults provide a basis for international comparisons. Studies in five European countries [22] revealed significant differences in carotenoid intake among countries, with dietary preferences such as carrots and spinach being key contributors. In contrast, studies from Singapore [23] and Brazil [15] indicated different dietary patterns and the influence of socioeconomic status on carotenoid sources. This juxtaposition of carotenoid intakes across studies in the U.S. and other countries underscores the global diversity in dietary patterns and data reporting. Understanding these differences is crucial for developing comprehensive nutritional policies and interventions tailored to specific dietary habits and socioeconomic contexts.

A major strength of this study is the use of a large nationally representative sample of the US adult population, allowing for a thorough analysis of total and individual carotenoid intake levels and major food sources among subgroups by gender, age, ethnicity, income, and lifestyle factors. Second, this study is the first analysis of the major food sources of total carotenoids and individual carotenoids among US adults over the previous decade. The consistency observed between the differences in major food sources of carotenoids over time and the trends in per capita food availability data suggests this research can offer valuable insights into understanding the long-term trends in carotenoid intake and consumption of carotenoid-rich foods. Nonetheless, it is imperative to acknowledge the limitations of the current study. Firstly, the estimation of dietary carotenoid intake relied on two 24 h dietary recalls, which may not accurately represent the participants’ habitual intake. Secondly, the study did not account for factors such as metabolism or bioavailability, potentially leading to estimates of dietary intake that may not align with biologically meaningful measures of exposure. To the best of the authors’ knowledge, this study is the first investigation into the alterations observed in the major food sources contributing to total carotenoids and individual carotenoids among adults in the United States throughout the previous decade. The consistency observed between variations in major food sources of carotenoids in this study, and the corresponding trends in per capita food availability data imparts significant insights for comprehending the temporal patterns in carotenoid intake and consumption of carotenoid-rich foods. Such comprehension of the long-term trends in carotenoid intake and the identification of dietary sources therein can assist in the identification of subgroups in need of interventions to improve carotenoid-rich fruit and vegetable intake and serve as a crucial foundation for future investigations concerning the potential associations between these changes and public health outcomes in the United States.

In conclusion, these findings indicate that carotenoid intake varies among subgroups of US adults, as did changes in the consumption of major food sources over the past 10 years. Our results warrant further studies investigating the consequences of the decreased tendencies of carotenoid intake on chronic disease risk, especially focusing on population subgroups exhibiting low or decreasing trends of carotenoid intake status.

## Figures and Tables

**Table 1:** Mean and Median intakes of individual and total carotenoids from diet among US adults aged 19 years and older by sociodemographic and lifestyle characteristics: NHANES 2009–2018 (n = 22,339).

Subgroups		α-Carotene (mcg/Day)	β-Carotene (mcg/Day)	β-Cryptoxanthin (mcg/Day)	Lycopene (mcg/Day)	Lutein + Zeaxanthin (mcg/Day)	Total (mcg/Day)
	*n*	Mean (Median)	Mean (Median)	Mean (Median)	Mean (Median)	Mean (Median)	Mean (Median)
All	22,339	422.0 (83.6)	2335.9 (1163.3)	85.7 (40.5)	5206.5 (2684.6)	1636.9 (872.2)	9687.1 (6821.4)
Gender							
Men	10,837	435.5 (74.7)	2288.2 (1098.3)	87.3 (41.9)	6101.2 (3240.6)	1599.4 (902.7)	10,512.0 (7371.4)
Women	11,502	409.0 (95.7)	2381.9 (1258.8)	84.2 (39.1)	4344.5 (2249.8)	1673.0 (850.1)	8892.6 (6426.2)
*p*-value ^1^		0.1734	0.1927	0.2835	<0.0001	0.1080	<0.0001
Age, y							
19–30	4263	348.2 (47.9)	1824.8 (763.6)	68.6 (31.8)	5456.8 (3031.0)	1374.8 (714.2)	9073.1 (6266.5)
31–50	7221	420.1 (71.6)	2342.8 (1070.0)	83.0 (39.3)	5520.7 (3020.3)	1705.1 (860.9)	10,072.0 (6915.7)
51–70	7518	460.9 (116.0)	2564.7 (1403.3)	95.2 (44.5)	5021.3 (2492.7)	1744.9 (964.7)	9887.1 (7205.9)
70+	3337	451.5 (154.3)	2596.6 (1510.9)	98.4 (49.3)	4325.0 (1859.6)	1600.7 (955.7)	9072.2 (6491.5)
*p*-value ^1^		<0.001	<0.0001	<0.0001	<0.0001	<0.01	0.5283
Men, y							
19–30	2136	360.7 (43.9)	1788.6 (735.6)	72.6 (33.0)	6396.4 (3617.0)	1327.1 (710.9)	9945.3 (6514.8)
31–50	3430	453.4 (68.6)	2362.4 (1079.6)	86.7 (41.7)	6534.2 (3637.5)	1689.5 (900.0)	11,126.0 (7681.9)
51–70	3636	468.8 (106.5)	2512.3 (1295.0)	94.7 (47.2)	5711.2 (2845.9)	1686.8 (1011.3)	10,474.0 (7736.5)
70+	1635	433.8 (148.8)	2431.5 (1412.0)	98.9 (48.8)	5179.2 (2242.9)	1615.8 (984.7)	9759.3 (6787.9)
*p*-value ^1^		0.0684	<0.0001	<0.001	<0.001	<0.01	0.8164
Women, y							
19–30	2127	334.3 (52.0)	1865.1 (791.5)	64.2 (29.9)	4411.2 (2483.5)	1427.8 (714.4)	8102.6 (6015.0)
31–50	3791	387.0 (76.8)	2323.4 (1069.1)	79.3 (36.7)	4516.8 (2448.2)	1720.6 (826.5)	9027.0 (6451.9)
51–70	3882	453.7 (129.6)	2613.3 (1494.6)	95.6 (43.1)	4381.7 (2145.5)	1798.8 (918.0)	9343.1 (6656.4)
70+	1702	464.9 (158.4)	2721.7 (1607.9)	98.1 (50.2)	3677.9 (1694.5)	1589.2 (923.2)	8551.8 (6095.1)
*p*-value ^1^		<0.0001	<0.0001	<0.0001	<0.05	<0.05	<0.05
Ethnicity							
White	9234	434.7 (86.9)	2385.0 (1235.6)	78.5 (38.7)	5358.9 (2811.2)	1640.4 (890.7)	9897.5 (7147.6)
Black	4914	290.3 (47.9)	2053.6 (761.7)	73.4 (35.6)	4211.1 (1784.5)	1682.6 (763.1)	8310.9 (5078.6)
Mexican-American	3161	379.0 (79.8)	1905.5 (968.0)	108.6 (57.8)	5688.5 (3460.8)	1271.2 (819.3)	9352.9 (6681.3)
Others	5030	495.6 (134.5)	2599.9 (1401.2)	115.2 (44.2)	5006.5 (2409.0)	1808.3 (942.0)	10,026.0 (7017.9)
*p*-value ^1^		0.3835	0.8971	<0.0001	0.1626	0.6419	0.5060
BMI ^2^							
BMI <18.5	587	528.0 (84.8)	2421.6 (1034.3)	70.8 (31.3)	4645.4 (2345.1)	1533.4 (751.6)	9199.2 (6479.3)
18.5 ≤ BMI < 25	5873	463.3 (96.4)	2618.8 (1303.7)	91.0 (40.2)	5542.5 (2664.0)	1873.1 (942.8)	10,589.0 (7419.3)
25 ≤ BMI < 30	7106	439.4 (95.2)	2446.3 (1275.0)	88.6 (42.6)	5152.5 (2769.0)	1719.6 (912.6)	9846.4 (7174.6)
30 ≤ BMI	8773	371.5 (70.3)	2035.9 (1016.3)	80.4 (39.4)	5043.9 (2605.1)	1404.7 (818.0)	8936.5 (6305.8)
*p*-value ^1^		<0.001	<0.0001	<0.05	0.1554	<0.0001	<0.0001
PIR ^3^							
<1.3	6542	331.0 (51.9)	1786.7 (737.8)	78.1 (33.8)	4895.5 (2491.6)	1307.7 (681.7)	8399.0 (5561.2)
1.3–1.85	2712	360.7 (66.0)	2064.8 (947.5)	84.6 (39.8)	4665.8 (2415.1)	1402.5 (791.5)	8578.4 (6010.2)
≥1.85	11,158	458.7 (104.6)	2560.9 (1387.7)	87.0 (42.4)	5468.3 (2844.8)	1799.8 (978.7)	10,375.0 (7556.7)
*p*-value ^1^		<0.0001	<0.0001	<0.05	<0.01	<0.0001	<0.0001
Alcohol consumption ^4^							
No	7958	442.0 (88.1)	2236.8 (1061.5)	90.3 (40.0)	4514.5 (2336.7)	1398.6 (810.5)	8682.1 (6052.6)
Moderate	7266	469.4 (107.6)	2678.5 (1465.2)	94.0 (44.6)	5589.5 (2761.4)	1887.6 (1029.2)	10,719.0 (7655.1)
Heavy	7115	358.9 (63.2)	2078.0 (973.6)	73.9 (36.5)	5391.6 (2932.0)	1583.2 (812.3)	9485.6 (6748.4)
*p*-value ^1^		<0.001	<0.05	<0.0001	<0.001	<0.05	<0.05
Smoking ^5^							
Never	12,474	456.8 (97.7)	2539.2 (1309.8)	92.1 (42.8)	5360.5 (2771.3)	1734.6 (914.5)	10,183.0 (7200.4)
Former	5353	462.9 (111.0)	2523.4 (1403.6)	89.7 (45.7)	5119.1 (2709.9)	1750.2 (987.3)	9945.3 (7397.7)
Current	3391	256.2 (38.4)	1399.9 (607.7)	58.3 (27.1)	4590.4 (2208.4)	1085.6 (602.4)	7390.4 (4907.9)
*p*-value ^1^		<0.0001	<0.0001	<0.0001	<0.01	<0.0001	<0.0001
Physical activity ^6^							
Light activity	8966	353.7 (67.0)	1897.6 (918.7)	76.1 (35.5)	4814.1 (2417.0)	1291.6 (741.4)	8433.0 (5854.8)
Moderate activity	3467	386.5 (83.9)	2185.4 (1154.4)	87.0 (38.1)	5244.1 (2757.9)	1470.6 (855.3)	9373.6 (6950.4)
Vigorous activity	9883	485.7 (99.5)	2717.9 (1377.3)	92.7 (45.1)	5493.8 (2888.1)	1952.3 (1005.5)	10,742.0 (7610.7)
*p*-value ^1^		<0.0001	<0.0001	<0.0001	<0.001	<0.0001	<0.0001
Supplement use							
No	11,692	350.8 (58.6)	1920.7 (880.5)	75.0 (34.0)	5255.6 (2757.4)	1381.7 (754.5)	8984.0 (6232.9)
Yes	10,647	492.3 (121.1)	2745.4 (1507.1)	96.3 (47.5)	5158.1 (2605.6)	1888.5 (1016.0)	10,381.0 (7486.0)
*p*-value ^1^		<0.0001	<0.0001	<0.0001	0.5813	<0.0001	<0.0001

^1^ *p*-value presented was calculated based on mean values; ^2^ Body Mass Index: kg/m^2^; ^3^ Ratio of the median family income over the poverty index. (PIR of ≤1.30 is required to be eligible for the Supplemental Nutrition Assistance Program); ^4^ Heavy alcohol consumption defined as consuming >2 drinks/day for men or >1 drink/day for women when consuming alcohol; moderate consumption defined as >0 and ≤2 drinks/day for men and >0 and ≤1 drinks/day for women when consuming alcohol; ^5^ Current smoking is defined as having smoked at least 100 cigarettes in their lifetime and smoked some days or every day; ^6^ Physical activity level, expressed using the MET score, was calculated by combining the intensity level of leisure time activities reported mean duration, and frequency.

**Table 2:** Trends in vitamin A adequacy rates among US adults aged 19 years and older by sociodemographic and lifestyle characteristics in the NHANES 2009–2018 (n = 22,339).

Subgroups	Vitamin A Adequacy Rate					
	2009–2010	2011–2012	2013–2014	2015–2016	2017–2018	
	*n*, %	*n*, %	*n*, %	*n*, %	*n*, %	*p*-Trend ^1^
All	1243, 27.8%	1049, 27.1%	1123, 27.7%	935, 25.3%	988, 25.5%	<0.0001
Gender						
Men	521, 24.0%	444, 25.7%	463, 24.5%	407, 22.8%	422, 21.9%	<0.0001
Women	722, 31.4%	605, 28.6%	660, 30.8%	528, 27.8%	566, 29.0%	<0.0001
*p*-value	<0.001	0.2042	<0.001	<0.01	<0.01	
Age, y						
19–30	208, 23.5%	197, 24.0%	198, 24.3%	154, 23.0%	151, 21.1%	<0.0001
31–50	402, 26.4%	319, 24.3%	367, 25.1%	303, 26.3%	295, 26.2%	<0.0001
51–70	394, 30.7%	368, 31.3%	379, 30.4%	300, 24.0%	354, 23.9%	<0.0001
70+	239, 32.2%	165, 30.1%	179, 34.4%	178, 29.9%	188, 35.9%	<0.0001
*p*-value	<0.01	<0.05	<0.01	0.1154	<0.01	
Ethnicity						
White	768, 31.7%	495, 30.9%	575, 30.7%	391, 28.3%	426, 27.5%	<0.0001
Black	181, 20.9%	238, 20.3%	179, 19.1%	175, 17.6%	210, 19.6%	<0.0001
Mexican-American	151, 16.0%	75, 18.1%	131, 21.7%	124, 20.9%	102, 21.7%	<0.0001
Others	143, 20.0%	241, 19.8%	238, 24.7%	245, 21.0%	250, 24.3%	<0.0001
*p*-value	<0.0001	<0.0001	<0.0001	<0.05	<0.0001	
PIR ^2^						
<1.3	314, 22.1%	304, 22.9%	289, 22.2%	220, 20.8%	213, 17.7%	<0.0001
1.3–1.85	127, 21.3%	113, 23.5%	115, 26.3%	127, 27.7%	127, 22.8%	<0.0001
≥1.85	705, 30.9%	556, 30.0%	633, 29.7%	505, 26.2%	541, 28.4%	<0.0001
*p*-value	<0.01	<0.001	<0.01	<0.05	<0.0001	

^1^ Cochran–Armitage test for trend was used to compare patterns of vitamin A adequacy rate over the 10 years. ^2^ Ratio of the median family income over the poverty index. (PIR of ≤1.30 is required to be eligible for Supplemental Nutrition Assistance Program).

**Table 3:** Trends in dietary vitamin A intake originated from retinol and carotenoids (provitamin A) among US adults aged 19 years and older by sociodemographic and lifestyle characteristics in the NHANES 2009–2018 (n = 22,339).

Subgroups	Provitamin A (mcg RAE/Day)						Retinol (mcg RAE/Day)					
	2009–2010	2011–2012	2013–2014	2015–2016	2017–2018		2009–2010	2011–2012	2013–2014	2015–2016	2017–2018	
	Mean (SE)	Mean (SE)	Mean (SE)	Mean (SE)	Mean (SE)	*p*-Trend	Mean (SE)	Mean (SE)	Mean (SE)	Mean (SE)	Mean (SE)	*p*-Trend
All	213.8 (4.9)	227.5 (14.0)	220.0 (7.8)	202.4 (9.2)	215.7 (11.3)	0.3991	451.1 (10.4)	436.7 (13.9)	430.2 (9.8)	434.1 (12.5)	418.7 (8.5)	0.0304
Gender												
Men	216.2 (10.7)	232.7 (20.3)	210.1 (6.4)	204.4 (13.4)	200.0 (13.1)	0.1404	495.0 (12.5)	502.0 (23.4)	482.1 (13.4)	476.4 (18.8)	454.6 (9.5)	0.012
Women	211.5 (7.3)	222.5 (14.7)	229.4 (12.5)	200.5 (6.4)	230.5 (11.7)	0.7579	409.0 (11.6)	373.9 (11.5)	380.5 (12.2)	392.2 (11.1)	384.5 (14.0)	0.4077
*p*-value	0.7635	0.6368	0.1240	0.6971	<0.01		<0.0001	<0.0001	<0.0001	<0.0001	<0.001	
Age, y												
19–30	159.0 (8.8)	169.0 (13.2)	172.6 (12.0)	176.9 (19.5)	169.1 (17.9)	0.5552	452.8 (19.8)	420.8 (19.2)	436.1 (16.4)	431.0 (22.0)	426.5 (16.3)	0.4088
31–50	212.5 (11.6)	212.9 (13.2)	229.4 (15.0)	194.8 (12.2)	232.4 (20.7)	0.676	442.1 (20.1)	416.7 (14.3)	402.0 (13.7)	434.3 (21.0)	413.6 (14.8)	0.4867
51–70	242.9 (6.8)	283.9 (31.5)	234.4 (8.2)	213.2 (11.8)	214.5 (15.8)	0.0119	457.0 (10.6)	455.1 (39.9)	429.5 (14.0)	402.2 (12.8)	392.8 (16.4)	0.0032
70+	242.4 (17.1)	222.3 (12.9)	240.8 (29.0)	236.1 (12.7)	252.5 (22.9)	0.5351	462.2 (17.5)	480.5 (26.1)	509.1 (16.7)	521.0 (60.7)	492.8 (22.2)	0.3228
*p*-value	<0.0001	<0.001	<0.01	<0.01	<0.05		0.6101	0.0963	<0.05	0.4044	0.3315	
Ethnicity												
White	224.8 (8.5)	242.2 (18.4)	220.1 (10.1)	200.1 (11.0)	213.5 (13.0)	0.0754	480.1 (8.4)	475.2 (17.5)	460.4 (11.8)	471.7 (16.1)	443.5 (9.6)	0.0243
Black	173.1 (11.9)	190.2 (22.7)	178.4 (11.2)	187.9 (12.9)	200.5 (19.1)	0.3179	426.5 (51.0)	357.0 (15.6)	359.8 (14.1)	312.7 (10.2)	353.4 (13.3)	0.0717
Mexican-American	170.7 (10.4)	152.3 (16.4)	188.4 (12.4)	176.3 (16.6)	203.9 (28.0)	0.2083	366.2 (21.4)	407.0 (20.3)	406.8 (20.2)	424.4 (23.2)	434.0 (39.3)	0.0889
Others	220.9 (19.8)	232.9 (17.8)	275.8 (23.5)	237.9 (16.8)	239.8 (12.1)	0.7807	365.5 (11.9)	332.3 (6.2)	361.2 (17.9)	365.9 (15.0)	363.9 (11.3)	0.2771
*p*-value	0.2398	0.1300	0.2205	0.1874	0.1493		<0.0001	<0.0001	<0.0001	<0.0001	<0.0001	
PIR ^1^												
<1.3	162.5 (5.9)	160.4 (13.3)	165.8 (12.6)	185.6 (13.1)	156.9 (12.5)	0.8119	426.1 (25.1)	400.5 (17.4)	399.3 (15.5)	387.1 (24.3)	402.0 (19.2)	0.4528
1.3–1.85	187.8 (12.6)	174.3 (13.5)	231.6 (23.3)	179.0 (19.7)	180.1 (16.6)	0.7502	428.7 (27.9)	451.0 (29.5)	448.4 (40.9)	418.1 (21.3)	445.2 (36.5)	0.9143
≥1.85	230.9 (6.5)	266.5 (18.8)	236.5 (9.9)	208.9 (10.8)	239.5 (12.6)	0.2744	463.6 (10.7)	454.5 (18.8)	439.3 (12.1)	439.2 (15.4)	426.2 (10.5)	0.018
*p*-value	<0.0001	<0.0001	<0.0001	<0.05	<0.0001		0.1764	0.0570	<0.05	<0.05	0.2362	

One mcg of RAE is equivalent to 1 mcg of retinol, 2 mcg of supplemental beta-carotene, 12 mcg of dietary beta-carotene, or 24 mcg of dietary alpha-carotene or beta-cryptoxanthin; ^1^ Ratio of the median family income over the poverty index. (PIR of ≤ 1.30 is required to be eligible for Supplemental Nutrition Assistance Program).

**Table 4:** Changes in Major Food Sources of Individual Carotenoids and Total Carotenoid in U.S. Adults Aged 19 and Older Over a Decade: NHANES 2009–2018.

Carotenoids		2009–2010 (n = 5084)				2017–2018 (n = 4160)			
	Rank	Food Group	Average Intake (mcg/Day)	Contribution	Cumulative Contribution	Food Group	Average Intake (mcg/Day)	Contribution	Cumulative Contribution
α-carotene	1	Carrot	301.7	71.5%	71.5%	Carrot	261.1	61.9%	61.9%
	2	Carrot juice	27.6	6.5%	78.0%	Tomatoes	15.1	3.6%	65.5%
	3	Mixed vegetables ^1^	20.3	4.8%	82.9%	Carrot juice	14.7	3.5%	68.9%
	4	Tomatoes	20.1	4.8%	87.6%	Mixed vegetables ^1^	9.1	2.1%	71.1%
	5	Pumpkin	7.0	1.7%	89.3%	Pumpkin	6.6	1.6%	72.7%
	6	Bananas	5.9	1.4%	90.7%	Bananas	5.8	1.4%	74.0%
	7	Squash	5.1	1.2%	91.9%	Beans ^2^	5.7	1.3%	75.4%
	8	Beans ^2^	4.6	1.1%	93.0%	Squash	4.3	1.0%	76.4%
	9	Vegetable/meat (or seafood) soup	3.8	0.9%	93.9%	Plantains	4.1	1.0%	77.4%
	10	Vegetable juice cocktail	3.5	0.8%	94.7%	Tangerines	3.9	0.9%	78.3%
β-carotene	1	Carrot	699.9	30.0%	30.0%	Carrot	607.9	26.0%	26.0%
	2	Sweet potato	213.3	9.1%	39.1%	Lettuce	322.4	13.8%	39.8%
	3	Spinach	177.4	7.6%	46.7%	Sweet potato	298.7	12.8%	52.6%
	4	Lettuce	140.8	6.0%	52.7%	Spinach	275.2	11.8%	64.4%
	5	Tomatoes	102.8	4.4%	57.1%	Tomatoes	75.8	3.2%	67.6%
	6	Melons	82.5	3.5%	60.7%	Melons	63.4	2.7%	70.4%
	7	Carrot juice	59.2	2.5%	63.2%	Tomato products ^3^	48.3	2.1%	72.4%
	8	Broccoli	56.6	2.4%	65.6%	Broccoli	35.3	1.5%	73.9%
	9	Tomato products ^3^	49.2	2.1%	67.7%	Pepper ^4^	32.7	1.4%	75.3%
	10	Mixed vegetables	43.7	1.9%	69.6%	Kale	32.1	1.4%	76.7%
β-cryptoxanthin	1	Orange juice	14.5	17.0%	17.0%	Tangerines	15.5	18.1%	18.1%
	2	Tangerines	8.1	9.4%	26.4%	Orange juice	9.4	10.9%	29.0%
	3	Oranges	7.8	9.1%	35.4%	Oranges	8.8	10.2%	39.2%
	4	Corn	7.5	8.8%	44.2%	Pepper ^3^	7.5	8.7%	47.9%
	5	Watermelon	6.3	7.4%	51.6%	Watermelon	6.3	7.4%	55.3%
	6	Persimmons	5.7	6.7%	58.3%	Chili	5.0	5.8%	61.1%
	7	Pepper ^4^	5.0	5.8%	64.1%	Corn	4.0	4.7%	65.8%
	8	Peaches	4.6	5.3%	69.4%	Carrot	3.2	3.8%	69.5%
	9	Pickles ^5^	3.5	4.0%	73.5%	Papaya	2.8	3.3%	72.8%
	10	Chili	3.4	3.9%	77.4%	Egg	2.7	3.2%	76.0%
Lycopene	1	Tomato products ^3^	1971.8	37.9%	37.9%	Tomato products ^3^	1971.8	37.9%	37.9%
	2	Tomatoes	677.9	13.0%	50.9%	Tomatoes	482.7	9.3%	47.1%
	3	Pizza	501.2	9.6%	60.5%	Salsa	456.6	8.8%	55.9%
	4	Watermelon	367.3	7.1%	67.6%	Pizza	424.8	8.2%	64.1%
	5	Catsup	348.2	6.7%	74.3%	Watermelon	366.4	7.0%	71.1%
	6	Vegetable juice cocktail	269.5	5.2%	79.4%	Catsup	320.5	6.2%	77.3%
	7	Salsa	257.0	4.9%	84.4%	Tomato Soup	166.8	3.2%	80.5%
	8	Tomato Soup	140.7	2.7%	87.1%	Other sauce ^6^	161.3	3.1%	83.6%
	9	Other sauce ^6^	127.0	2.4%	89.5%	Tomato juice	100.4	1.9%	85.5%
	10	Tomato juice	120.9	2.3%	91.8%	Pasta ^7^	86.6	1.7%	87.2%
Lutein + zeaxanthin	1	Spinach	358.4	21.9%	21.9%	Spinach	592.4	36.2%	36.2%
	2	Egg	102.1	6.2%	28.1%	Egg	148.9	9.1%	45.3%
	3	Lettuce	84.3	5.1%	33.3%	Lettuce	134.6	8.2%	53.5%
	4	Broccoli	83.2	5.1%	38.4%	Broccoli	100.4	6.1%	59.6%
	5	Squash	74.4	4.5%	42.9%	Squash	46.8	2.9%	62.5%
	6	Chicory greens	62.7	3.8%	46.7%	Kale	44.5	2.7%	65.2%
	7	Corn	60.4	3.7%	50.4%	Corn	40.5	2.5%	67.7%
	8	Collards	52.4	3.2%	53.6%	Beans ^2^	33.2	2.0%	69.7%
	9	Kale	41.1	2.5%	56.1%	Carrot	27.3	1.7%	71.4%
	10	Beans ^2^	38.7	2.4%	58.5%	Cereals ^8^	27.1	1.7%	73.0%
Total	1	Tomato products ^3^	2039.0	21.0%	21.0%	Tomato products ^3^	2003.9	20.7%	20.7%
	2	Carrot	1035.7	10.7%	31.7%	Carrot	899.6	9.3%	30.0%
	3	Tomatoes	830.4	8.6%	40.3%	Spinach	867.6	9.0%	38.9%
	4	Spinach	535.9	5.5%	45.8%	Tomatoes	595.0	6.1%	45.1%
	5	Pizza	534.1	5.5%	51.4%	Salsa	497.0	5.1%	50.2%
	6	Watermelon	398.8	4.1%	55.5%	Lettuce	457.2	4.7%	54.9%
	7	Catsup	361.9	3.7%	59.2%	Pizza	452.7	4.7%	59.6%
	8	Vegetable juice cocktail	297.8	3.1%	62.3%	Watermelon	397.8	4.1%	63.7%
	9	Salsa	279.6	2.9%	65.2%	Catsup	333.2	3.4%	67.1%
	10	Lettuce	225.7	2.3%	67.5%	Sweet potato	299.4	3.1%	70.2%
	11	Sweet potato	213.8	2.2%	69.7%	Tomato Soup	171.8	1.8%	72.0%
	12	Tomato Soup	144.7	1.5%	71.2%	Other sauce ^6^	171.1	1.8%	73.8%
	13	Broccoli	140.5	1.4%	72.7%	Egg	151.8	1.6%	75.3%
	14	Other sauce ^6^	134.6	1.4%	74.0%	Broccoli	137.6	1.4%	76.8%
	15	Tomato juice	125.3	1.3%	75.3%	Pasta ^7^	104.2	1.1%	77.8%
	16	Squash	105.8	1.1%	76.4%	Tomato juice	104.0	1.1%	78.9%
	17	Egg	104.8	1.1%	77.5%	Vegetable juice cocktail	84.2	0.9%	79.8%
	18	Collards	89.9	0.9%	78.4%	Chili	79.3	0.8%	80.6%
	19	Carrot juice	88.9	0.9%	79.4%	Kale	77.3	0.8%	81.4%
	20	Melons	84.5	0.9%	80.2%	Squash	72.0	0.7%	82.1%

^1^ Mixed vegetables usually are made up of an assortment of different vegetables, typically including peas, carrots, corn, green beans, lima beans; ^2^ Beans included peas, lentils, cowpeas and lupins; ^3^ Tomato products included pasta and spaghetti sauce, marinara sauce, tomato paste, and tomato puree; ^4^ Pepper included pimento, but excluded chili powder and chili; ^5^ Pickles included pickle relish; ^6^ Other sauce included barbecue sauce, steak sauce, and cocktail sauce, excluding gravy, salsa, pepper, guava, and chili; ^7^ Pasta included lasagna, spaghetti, ravioli, tortellini, and macaroni; ^8^ Cereals included cereals ready-to-eat and cereal bar.

## Data Availability

Data were obtained from the Centers for Disease Control and Prevention National Center for Health Statistics at https://www.cdc.gov/nchs/nhanes/index.htm (accessed on 19 October 2023).

## Supplementary Materials

The following supporting information can be downloaded at https://www.mdpi.com/article/10.3390/metabo14010013/s1, Table S1: Average Energy-Adjusted Intake of Total Carotenoids and Individual Carotenoids from Diet among US Adults aged 19 and older by Sociodemographic and Lifestyle Characteristics: NHANES 2009–2018; Table S2: Daily Dietary Intake of Individual Carotenoids (mcg) of US adults aged 19 and older and by sociodemographic characteristics in the NHANES 2009–2018; Table S3: Average Provitamin A Intake from Diet among US Adults aged 19 and older by Sociodemographic and Lifestyle Characteristics: NHANES 2009–2018.

## References

[1] Stahl W., Sies H. (2003). Antioxidant activity of carotenoids. Mol. Asp. Med..

[2] Li Z., Chen J., Zhang D. (2019). Association between dietary carotenoid intakes and hypertension in adults: National Health and Nutrition Examination Survey 2007–2014. J. Hypertens..

[3] Sluijs I., Cadier E., Beulens J.W., van der A.D., Spijkerman A.M., van der Schouw Y.T. (2015). Dietary intake of carotenoids and risk of type 2 diabetes. Nutr. Metab. Cardiovasc. Dis..

[4] Arab L., Steck S. (2000). Lycopene and cardiovascular disease. Am. J. Clin. Nutr..

[5] Kritchevsky S.B., Tell G.S., Shimakawa T., Dennis B., Li R., Kohlmeier L., Steere E., Heiss G. (1998). Provitamin A carotenoid intake and carotid artery plaques: The Atherosclerosis Risk in Communities Study. Am. J. Clin. Nutr..

[6] Leoncini E., Edefonti V., Hashibe M., Parpinel M., Cadoni G., Ferraroni M., Serraino D., Matsuo K., Olshan A.F., Zevallos J.P. (2016). Carotenoid intake and head and neck cancer: A pooled analysis in the International Head and Neck Cancer Epidemiology Consortium. Eur. J. Epidemiol..

[7] Wu S., Liu Y., Michalek J.E., Mesa R.A., Parma D.L., Rodriguez R., Mansour A.M., Svatek R., Tucker T.C., Ramirez A.G. (2020). Carotenoid Intake and Circulating Carotenoids Are Inversely Associated with the Risk of Bladder Cancer: A Dose-Response Meta-analysis. Adv. Nutr..

[8] Jung S., Wu K., Giovannucci E., Spiegelman D., Willett W.C., Smith-Warner S.A. (2013). Carotenoid intake and risk of colorectal adenomas in a cohort of male health professionals. Cancer Causes Control.

[9] Van Hoang D., Pham N.M., Lee A.H., Tran D.N., Binns C.W. (2018). Dietary Carotenoid Intakes and Prostate Cancer Risk: A Case-Control Study from Vietnam. Nutrients.

[10] Nkondjock A., Ghadirian P., Johnson K.C., Krewski D., Canadian Cancer Registries Epidemiology Research G. (2005). Dietary intake of lycopene is associated with reduced pancreatic cancer risk. J. Nutr..

[11] Bernstein P.S., Li B., Vachali P.P., Gorusupudi A., Shyam R., Henriksen B.S., Nolan J.M. (2016). Lutein, zeaxanthin, and meso-zeaxanthin: The basic and clinical science underlying carotenoid-based nutritional interventions against ocular disease. Prog. Retin. Eye Res..

[12] Hughes D.A. (2001). Dietary carotenoids and human immune function. Nutrition.

[13] Pattison D.J., Symmons D.P., Lunt M., Welch A., Bingham S.A., Day N.E., Silman A.J. (2005). Dietary beta-cryptoxanthin and inflammatory polyarthritis: Results from a population-based prospective study. Am. J. Clin. Nutr..

[14] Davinelli S., Ali S., Solfrizzi V., Scapagnini G., Corbi G. (2021). Carotenoids and Cognitive Outcomes: A Meta-Analysis of Randomized Intervention Trials. Antioxidants.

[15] Carnauba R.A., Sarti F.M., Hassimotto N.M.A., Lajolo F.M. (2022). Assessment of dietary intake of bioactive food compounds according to income level in the Brazilian population. Br. J. Nutr..

[16] Estevez-Santiago R., Beltran-de-Miguel B., Olmedilla-Alonso B. (2016). Assessment of dietary lutein, zeaxanthin and lycopene intakes and sources in the Spanish survey of dietary intake (2009–2010). Int. J. Food Sci. Nutr..

[17] Manzi F., Flood V., Webb K., Mitchell P. (2002). The intake of carotenoids in an older Australian population: The Blue Mountains Eye Study. Public. Health Nutr..

[18] Amiot M.J., Latge C., Plumey L., Raynal S. (2021). Intake Estimation of Phytochemicals in a French Well-Balanced Diet. Nutrients.

[19] Han S., Wu L., Wang W., Li N., Wu X. (2019). Trends in Dietary Nutrients by Demographic Characteristics and BMI among US Adults, 2003–2016. Nutrients.

[20] Nebeling L.C., Forman M.R., Graubard B.I., Snyder R.A. (1997). The impact of lifestyle characteristics on carotenoid intake in the United States: The 1987 National Health Interview Survey. Am J Public Health.

[21] Bentley J.U.S. (2017). Trends in Food Availability and a Dietary Assessment of Loss-Adjusted Food Availability, 1970–2014.

[22] O’Neill M.E., Carroll Y., Corridan B., Olmedilla B., Granado F., Blanco I., Van den Berg H., Hininger I., Rousell A.M., Chopra M. (2001). A European carotenoid database to assess carotenoid intakes and its use in a five-country comparative study. Br. J. Nutr..

[23] Toh D.W.K., Loh W.W., Sutanto C.N., Yao Y., Kim J.E. (2021). Skin carotenoid status and plasma carotenoids: Biomarkers of dietary carotenoids, fruits and vegetables for middle-aged and older Singaporean adults. Br. J. Nutr..

[24] Kiani A.K., Dhuli K., Donato K., Aquilanti B., Velluti V., Matera G., Iaconelli A., Connelly S.T., Bellinato F., Gisondi P. (2022). Main nutritional deficiencies. J. Prev. Med. Hyg..

[25] Stephensen C.B. (2001). Vitamin A, infection, and immune function. Annu. Rev. Nutr..

[26] Semba R.D. (1994). Vitamin A, immunity, and infection. Clin. Infect. Dis..

[27] Correia L.L., Rocha H.A.L., Campos J.S., Silva A.C.E., Silveira D., Machado M.M.T., Leite A.J.M., Cunha A. (2019). Interaction between vitamin A supplementation and chronic malnutrition on child development. Cien. Saude. Colet..

[28] Gilbert C. (2013). The eye signs of vitamin A deficiency. Community Eye Health.

[29] West K.P. (2003). Vitamin A deficiency disorders in children and women. Food Nutr. Bull..

[30] Latham M.C. (1997). Human Nutrition in the Developing World.

[31] Division N. (2002). Human Vitamin and Mineral Requirements.

[32] National Institutes of Health (NIH) Office of Dietary Supplements (ODS) Vitamin A and Carotenoids. https://ods.od.nih.gov/factsheets/VitaminA-HealthProfessional/.

[33] U.S. Department of Agriculture, A.R.S (2012). USDA Food and Nutrient Database for Dietary Studies, 5.0.

[34] U.S. Department of Agriculture, A.R.S (2014). USDA Food and Nutrient Database for Dietary Studies 2011–2012.

[35] U.S. Department of Agriculture, A.R.S (2016). USDA Food and Nutrient Database for Dietary Studies 2013–2014.

[36] U.S. Department of Agriculture, A.R.S (2018). USDA Food and Nutrient Database for Dietary Studies 2015–2016.

[37] U.S. Department of Agriculture, A.R.S (2020). USDA Food and Nutrient Database for Dietary Studies 2017–2018.

[38] Haytowitz D.B., Ahuja J.K.C., Wu X., Somanchi M., Nickle M., Nguyen Q.A., Roseland J.M., Williams J.R., Patterson K.Y., Li Y. (2019). USDA National Nutrient Database for Standard Reference, Legacy Release.

[39] Krauss R.M., Eckel R.H., Howard B., Appel L.J., Daniels S.R., Deckelbaum R.J., Erdman J.W., Kris-Etherton P., Goldberg I.J., Kotchen T.A. (2001). Revision 2000: A statement for healthcare professionals from the Nutrition Committee of the American Heart Association. J. Nutr..

[40] Ainsworth B.E., Haskell W.L., Herrmann S.D., Meckes N., Bassett D.R., Tudor-Locke C., Greer J.L., Vezina J., Whitt-Glover M.C., Leon A.S. (2011). 2011 Compendium of Physical Activities: A second update of codes and MET values. Med. Sci. Sports Exerc..

[41] Krebs-Smith S.M., Cook A., Subar A.F., Cleveland L., Friday J. (1995). US adults’ fruit and vegetable intakes, 1989 to 1991: A revised baseline for the Healthy People 2000 objective. Am. J. Public. Health.

[42] Hoy M.K., Goldman J.D., Moshfegh A.J. (2017). Differences in fruit and vegetable intake of U.S. adults by sociodemographic characteristics evaluated by two methods. J. Food Compos. Anal..

[43] Subar A.F., Heimendinger J., Patterson B.H., Krebs-Smith S.M., Pivonka E., Kessler R. (1995). Fruit and vegetable intake in the United States: The baseline survey of the Five A Day for Better Health Program. Am. J. Health Promot..

[44] Garriguet D. (2009). Diet quality in Canada. Health Rep..

[45] Beydoun M.A., Wang Y. (2008). How do socio-economic status, perceived economic barriers and nutritional benefits affect quality of dietary intake among US adults?. Eur. J. Clin. Nutr..

[46] Institute of Medicine (US) Panel on Dietary Antioxidants and Related Compounds (2000). Dietary Reference Intakes for Vitamin C, Vitamin E, Selenium, and Carotenoids.

[47] Bohm V., Lietz G., Olmedilla-Alonso B., Phelan D., Reboul E., Banati D., Borel P., Corte-Real J., de Lera A.R., Desmarchelier C. (2021). From carotenoid intake to carotenoid blood and tissue concentrations—Implications for dietary intake recommendations. Nutr. Rev..

[48] Economic Research Service (ERS), U.S.D.o.A.U (2001). Food Availability (Per Capita) Data System.

